# Mortality, Enzymatic Antioxidant Activity and Gene Expression of Cabbage Aphid (*Brevicoryne brassicae* L.) in Response to *Trichoderma longibrachiatum* T6

**DOI:** 10.3389/fphys.2022.901115

**Published:** 2022-07-19

**Authors:** Rehan Inayat, Aroosa Khurshid, Solomon Boamah, Shuwu Zhang, Bingliang Xu

**Affiliations:** College of Plant Protection, Gansu Agricultural University/Biocontrol Engineering Laboratory of Crop Diseases and Pests of Gansu Province, Lanzhou, China

**Keywords:** mortality, antioxidant enzyme, *Brevicoryne brassicae*, gene expression, *Trichoderma* spp.

## Abstract

Aphids are one of the most common insect pests in greenhouse and field crops worldwide, causing significant crop yields and economic losses. The objective of this study was to determine the mortality, enzymatic antioxidant activity and gene expression of cabbage aphids (*Brevicoryne brassicae* L.) in response to *Trichoderma longibrachiatum* T6 (T6) at different time points from Day 1 to 7 after inoculation. Our results showed that the highest mortality of *B. brassicae* was observed on Day 7 at a concentration of 1 × 10^8^ spores ml^−1^ (73.31%) after inoculation with T6 compared with the control on Day 7 (11.51%). The activities of the enzymes superoxide dismutase (SOD), peroxidase (POD) and catalase (CAT), ascorbate peroxidase (APX), glutathione peroxidase (GPX) and glutathione S-transferase (GST) were increased by 52.98%, 44.77%, 48.26%, 49.39%, 45.13% and 39.67%, respectively on Day 3 after inoculation with T6 compared to the control. Howerver increased days post treatment (dpt) decreased the activities of SOD*,* POD*,* CAT*,* APX*,* GPX and GST enzymes by 20.79%, 21.05%, 13.75%, 20.45%, 25.38%, and 19.76% repectively on Day 7 compared to control. The transcript levels of *SOD*, *POD*, *CAT*, *GPX*, and *GST* genes were increased by 10.87, 9.87, 12.77, 6.22 and 4.07 respectively at Day 3 after inoculation with T6 in comparison to the control. However, the *SOD*, *POD*, *CAT*, *GPX*, and *GST* transcription levels decreased by 0.43, 0.44, 0.35, 0.52 and 0.47 respectively, compared to control at Day 7*.* Our results suggest that the T6 strain has a potential effect on the antioxidant activity and mortality of *B. brassicae* and therefore could be used as a natural biocontrol agent against *B. brassicae* in the future.

## Introduction

*Brevicoryne brassicae* L. (Hemiptera: Aphididae), the cabbage aphid, is a major pest endemic to Europe but now occurring internationally (Kessing and Mau, 1991; Munthali, 2009). The Brassicaceae family has been severely damaged in many areas, including Canada, Netherlands, South Africa, India, and China (Carter and Sorenson, 2013). It causes significant damage to commercially important crops such as cauliflower (*Brassica oleracea* var. *botrytis*), canola (*Brassica napus*), brussels sprouts (*Brassica oleracea* var. *gemmifera*), broccoli (*Brassica oleracea* var. *italica*), white and black mustard (*Brassica nigra*), toria (*Brassica campestris*), Chinese cabbage (*Brassica rapa* subsp. *pekinensis*), and other crops such as kale (*Brassica oleracea*) (Kessing and Mau, 1991). It also causes yellowing and wilting and acts as a vector of several viral infections in cruciferous crops such as turnip mosaic virus and cauliflower mosaic virus (Chivasa et al., 2002; Opfer and Mcgranth, 2013). In severe conditions, plants become infested with aphids, and as a result of the production of honey dew from aphid cornicles, formation of black mold takes place covering the entire leaf surface, which can eventually lead to plant death (Blackman and Eastop, 2008; Griffin and Williamson, 2012; Jabbar and Sasdoon, 2019). However, the increasing use of chemicals over time has detrimental effects on the biotic complex of nature and affects plants, animals, and humans (Baweja et al., 2020). The focus on alternative pest control methods has gained attention in the pest management and currently, the application of cultural and biological control strategies can help reduce aphid infestations and pesticide use while maintaining yield and produce quality. As a result of recent developments in biotechnology, there has been renewed interest in the use of entomopathogenic fungi as biological control agents (BCAs), and further progress is being made in the development and production of these agents (Khachatourians, 1986). Entomopathogenic fungi are used for healthy and natural control of insects and arthropods such as grasshoppers, mosquitoes, aphids, and thrips (Sparagano and Giangaspero, 2011). A number of fungi, including *Paecilomyces fumosoroseus*, *Metarhizium* spp., *Lecanicillium lecanii*, *Buveria bassiana*, *Hirsutella thompsonii*, and *Nomuraea rileyi*, have recently been shown to provide protection against whiteflies, thrips, aphids, beetles, grasshoppers, grasshopper worms, and grasses (Charnley, 1984; Srinivasan, 2008; Mnyone et al., 2009; Lwetiojera et al., 2010).

*Trichoderma* species as biocontrol agents against plant-infecting pathogens has been documented since the 1920s. There is a long history of fungal biocontrol research and the use of myco-biocontrol agents such as *Trichoderma* has focused on enhancing their ability to directly attack a fungal or bacterial plant pathogen or even live insects through competition for resources and space, parasitism, and antimicrobial production (Harman, 2006; Harman et al., 2008; Boamah et al., 2021b). *Trichoderma* also has a very effective biocontrol mechanism to suppress the development of pathogens such as mycoparasitism, antibiosis and competition (Chamzurni et al., 2013; Boamah et al., 2021a). In previous studies, *T. longibrachiatum* T6 was reported to have higher potential parasitic and lethal activity against *Heterodera avenae* (Zhang et al., 2014). In addition, *T. longibrachiatum* was an effective entomopathogenic agent for the control of the insect *Leucinodes orbonalis*, an important pest of brinjal, where field application of a formulated product of this strain showed efficacy similar to that of the pesticide malathion (Ghosh and Pal, 2016). Lastly, *T. longibrachiatum* revealed a mortality of 73% against *Bemisia tabaci* nymphs at 4 × 10^8^ conidia ml^−1^ after 144 h of exposure, while the maximum adult mortality under the same conditions was 40% (Anwar et al., 2016).

The performance of an insect on plants is influenced by the balance between the production and elimination of reactive oxygen species (ROS) (Krishnan and Sehnal, 2006). The antioxidant enzyme system of aphids and other herbivorous insects reduces the levels of ROS (Durak et al., 2021). The antioxidant system of an insect consists of several antioxidant enzymes (superoxide dismutase-SOD, catalase-CAT, peroxidase-POD, glutathione transferase-GST, glutathione reductase-GR, ascorbate oxidase-AO, ascorbate peroxidase-APX). These enzymes play an essential role in combating oxidative stress (Valko et al., 2006). Jia et al. (2016) reported that the SOD and POD enzyme activities of *Locusta migratoria* nymphs were increased at the first period, and decreased later after treated with *M. anisopliae*. However, the determination of mortality and enzymatic antioxidant activity and gene expression of *B. Brassicae* in response to *T. longibrachiatum* T6 (T6) has never been investigated in previous studies. In the present study, the aims were to determine the toxic and parasitic effect of T6 on the *B. brassicae* at different time points after inoculation, and determine the enzymatic antioxidant activity and gene expression of *B. brassicae* in response T6 inoculation.

## Materials and Methods

### Fungal Preparation

*Trichoderma longibrachiatum* T6 was obtained from the Plant Pathology laboratory of Gansu Agricultural University and was inoculated in freshly prepared potato dextrose agar (PDA) media. PDA media was incubated in an artificial growth chamber for 6 days 25°C and 16: 8 h (light: dark). After 6 days of incubation, the spores of freshly grown T6 were collected and the spores suspension was prepared according to the method decribeed by Zhang et al. (2014). The concentration of the spore suspension was determined with a hemacytometer (Hausser Scientific™ Bright-Line™ Counting Chamber, China), and the final concentration was prepared and adjusted to 1 × 10^3^, 1 × 10^4^, 1 × 10^5^, 1 × 10^6^, 1 × 10^7^ and 1 × 10^8^ spores ml^−1^ and 0.01% Tween 80 was added to the suspensions for further use.

### Cabbage Cultivation

The super-elite variety of cabbage was grown in seedling trays with compost in a greenhouse (photoperiod-16: 8 h (light: dark), temperature-26 ± 1°C, relative humidity - 70 ± 5%). The seedling tray was kept moist until the seeds germinated and were ready for transplanting. Transplanting occurred when the seedlings had developed at least two mature leaves and were transplanted into larger plastic pots. These pots were watered every 2 days to keep the cabbage fresh for aphid culture. The transplanted seedlings were kept in insect cages to minimize the risk of contamination by other pests in the greenhouse.

### Aphid Cultivation

Adults of *B. brassicae* were collected from the experimental field in Gansu Agricultural University and reared over home-grown cabbage in the insect rearing room. The aphids were reared on cabbage and maintained under controlled conditions in a climate chamber [photoperiod-14: 10 h (light: dark), temperature-27 ± 1°C, relative humidity-60 ± 5%].

### Determination of the Toxic and Parasitic Effect of *T. longibrachiatum* T6 on *B. brassicae*

Aphids were treated with different concentrations of T6 spore suspension (1 × 10^3^, 1 × 10^4^, 1 × 10^5^, 1 × 10^6^, 1 × 10^7^ and 1 × 10^8^ spores ml^−1^)using the detached leaf method. The fresh leaves of cabbage were washed three times with distilled water and air dried for the aphid inoculation. The leaves were placed on the (9 cm diameter) plastic Petri dishes. The wet cotton wrapped around the detached part of the leaves was used only to provide water to the leaves to maintain relative humidity during the test. A group of 30 adult aphids were dipped in each concentration of T6 spores suspension one by one for 3–5 s using a sterilized camel hair brush and then placed on each leaf. All treated aphids were checked by touching and moving them with the brush to ensure that they were still alive and had not died from being immersed in the spore suspension. The Petri plates were placed in an incubator operated at 25 ± 2°C and 60 ± 5% relative humidity with a 16:8 h (light: dark), after making sure they were all alive and suitable for bioassay studies. The adult aphids were treated with sterilized water and 0.01% Tween 80 were used as the control. The experiment was conducted with three replicates of each treatment, and the aphid mortality was recorded daily for Day 1–7 after inoculation.

### Determination of ROS Scavenging Antioxidant Enzymes Activity of *B. brassicae* After Inoculation

Aphids (30 individuals) were homogenized in a phosphate buffer (0.1 M, pH 7.0) at 25°C with a homogenizer after exposure to different concentrations of T6 and control from Days 1–7. The enzyme activities were determined using commercial assay kits (Beijing Solarbio Science and Technology Company Limited, China) according to the manufacturer’s protocol. Briefly, the homogenate was centrifuged at 8,000 g for 10 min at 4°C. The supernatant was used to determine the enzymatic activity of superoxide dismutase (SOD, EC1.15.1.1), peroxidase (POD, EC1.11.1.7), catalase (CAT, EC1.11.1.6), glutathione S-transferase (GST, BC0355), ascorbate peroxidase (APX, BC0220), and glutathione peroxidase (GPX, BC1195) using a spectrophotometer (SP -756P, Shanghai, China). Enzyme activities were expressed as units of mg per fresh weight of aphids (U mg^−1^ FW). The activity of SOD, POD, CAT, GST APX, GPX was measured as the amount of crude enzyme extract at 560, 470, 240, 340, 290, 480 nm.

### Oxidant Contents of *B. brassicae* Measurement After Inoculation

The contents of malondialdehyde (MDA) and H_2_O_2_ was measured according to the manufacturer’s protocol using the test kits provided (Solarbio, China). Briefly, MDA samples were prepared from 0.2 g of aphid tissue homogenized in 0.1 M potassium phosphate buffer (pH 7.0). The homogenate was centrifuged at 12,000 g for 20 min at room temperature, and then 0.5 ml of the supernatant was added to 2 ml of 20% trichloroacetic acid (TCA) containing 0.5% thiobarbituric acid (TBA). The mixture was incubated at 95°C for 30 min and then the reaction was stopped by cooling the tubes in an ice water bath. Another centrifugation was performed at 10,000 g for 10 min. The absorbance of the MDA sample was measured at three different wavelengths (450, 532, and 600 nm) and that of H_2_O_2_ at 415 nm using a spectrophotometer (EPOCH2 Plate Reader, BioTek, America). The content of MDA and H_2_O_2_ was expressed in μmol g^−1^ FW.

### Antioxidant Enzymes Genes Expression of *B. brassicae* Measurement After Inoculation

The gene expression level of ROS scavenging enzymes SOD, POD, CAT, GPX and GST genes in *B. brassicae* were determined after inoculation with T6 spores suspension at different time points after inoculation. Total RNA was extracted using the Total RNA extraction kit from 100 mg of treated and untreated aphids (Solarbio Biotechnology, Beijing, China). The concentration of RNA was measured using a Nano-Drop spectrophotometer at absorbances of 260 and 280 nm. The cDNA was synthesized using M5 Hiper RT cDNA Synthesis Kit (Mei5 Biotechnology, Beijing, China) according to manufacturer’s protocol. In the RT-qPCR studies, primer sequences were constructed using the software Primer Express 3.0 based on the NCBI target gene sequences and listed in Table 1. The key information of the genes has been provided as supplemental material (Supplementary Table S1). These primers were used in the analysis of the RT-qPCR data. The actin gene of aphid was used as an internal control (Khurshid et al., 2021). The relative expression level was quantified using the comparative 2^−ΔΔCt^ method (Livak and Schmittgen, 2001).

**TABLE 1:** Primers used in the qRT- PCR.

Gene name	Primer sequences (5′-3′)	Gene ID	Organism
Actin depolymerizing factor	F:ATTTTCCAGCGGCAAACGTC	112679496	*Sipha flava*
	R: GTT​CTT​CCA​CAA​CGT​AAT​CGT​TT		
Catalase	F: TAT​ACA​GTG​GCG​TGG​TCT​GC	100159682	*Acyrthosiphon pisum*
	R: ATC​TCC​CGG​ATG​GAT​GGT​CA		
Glutathione peroxidase 2	F: GCC​GTT​AAG​GGG​CAG​ATT​CA	112679719	*Sipha flava*
	R: TCC​GCT​TGG​ATT​AGG​CTT​GG		
Glutathione S-transferase	F: TAG​CTG​GTA​GCG​ATG​ATT​GGG	107169986	*Diuraphis noxia*
	R: TAT​TGA​GAC​ACG​GGC​ACC​G		
Peroxidase	F: CCG​GAA​AGA​CAC​CGA​AGA​GT	111033629	*Myzus persicae*
	R: GCA​CAG​TTA​GGG​AAC​GGA​TG		
Superoxide dismutase	F: TGC​TAT​AGG​ACA​GGG​TGC​AG	113551032	*Rhopalosiphum maidis*
	R: AAG​CTC​CTG​GTT​CAT​CGG​TG		

### Data Analysis

The data was subject to one-way ANOVA using the SPSS Version 26.0 (SPSS, Inc., Chicago, IL, USA). Each treatment had three replicates. Treatment effects were determined using Duncan’s multiple range test and the significances were expressed at *p* < 0.05.

## Results

### Effect of *T. longibrachiatum* T6 on the Mortality of *B. brassicae*

**FIGURE 1:**
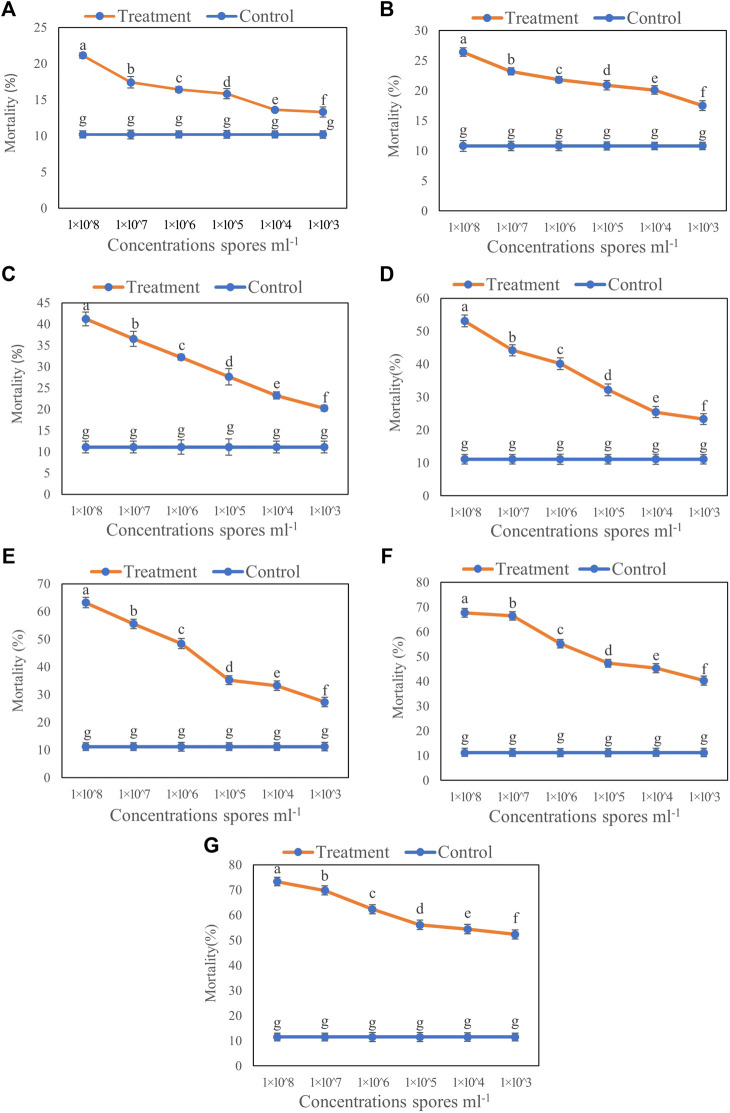
Mortality of *B. brassicae* after treated with different concentrations of *T. longibrachiatum* T6 spore suspension at **(A)** Day 1, **(B)** Day 2, **(C)** Day 3, **(D)** Day 4, **(E)** Day 5, **(F)** Day 6 and **(G)** Day 7 by the dipping method. Different small letters represent the significant difference at the *p* < 0.05 level by Duncan’s new multiple range test. The line bars represent means ± standard error.

The results of inoculation by the aphid dip method showed that increased concentration of T6 spore suspension significantly increased the mortality of *B. brassicae* (Figure 1). Compared to the control, the highest mortality of *B. brassicae* was observed at 7 days after inoculation with 1 × 10^8^ spores ml^−1^ T6 spore suspension. The lowest mortality was observed with 1 × 10^3^ spores ml^−1^ T6 spore suspension at Day 1 after inoculation (Figure 1). Increasing the number of days after T6 spore suspension inoculation significantly increased aphid mortality. The mortality of *B. brassicae* was 21.12% (Figure 1A), 26.41% (Figure 1B), 41.21% (Figure 1C), 53.12% (Figure 1D), 63.21% (Figure 1E), 67.72% (Figure 1F), and 73.31% (Figure 1G) from Days 1 to 7 after inoculation with T6 at 1 × 10^8^ spores ml^−1^, respectively.

### Parasitic Effect of *T. longibrachiatum* T6 on *B. brassicae*

**FIGURE 2:**
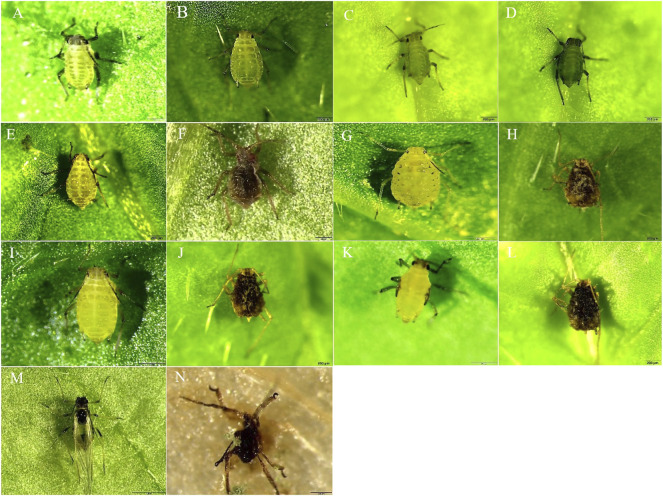
The parasitic effect of *T. longibrachiatum* T6 spore suspension on *B. brassicae* from Day 1 to day 7 post-inoculation. **(A,B)** shows Day 1 control and T6-treated *B. brassicae*; **(C,D)** shows Day 2 control and T6-treated *B. brassicae*; **(E,F)** shows Day 3 control and T6-treated *B. brassicae*; **(G,H)** shows Day 4 control and T6-treated *B. brassicae*; **(I,J)** shows Day 5 control and T6-treated *B. brassicae*; **(K,L)** shows Day 6 control and T6-treated *B. brassicae*; **(M,N)** shows Day 7 control and T6-treated *B. brassicae*.

Our results showed that the strain of T6 had a significant parasitic effect on *B. brassicae* from Day 1 to 7 after inoculation. On Day 1, *B. brassicae* inoculated with T6 (Figure 2B) showed no visible change in morphology compared to the control (Figure 2A). On Day 2, the color of treated aphids (Figure 2D) began to change from light green to dark green compared to the control (Figure 2C). On Day 3, the surface of *B. brassicae* was parasitized by the mycelium of T6 and the color changed from dark green to brown (Figure 2F) compared to the control group, which showed normal growth and development (Figure 2E). On Day 4 (Figure 2H), the mycelium of T6 colonized the entire body of the dead aphids compared to the control group (Figure 2G). On Day 5–6 (Figures 2J–L), the color became darker and the aphid body surface deformed compared to the control (Figures 2I–K). On day 7, the T6 spores parasited on different body parts of the dead aphids (Figure 2N) compared to the control, which showed fully grown winged adult (Figure 2M).

### Effect of *T. longibrachiatum* T6 on the Antioxidant Enzyme Activities of *B. brassicae*

**FIGURE 3:**
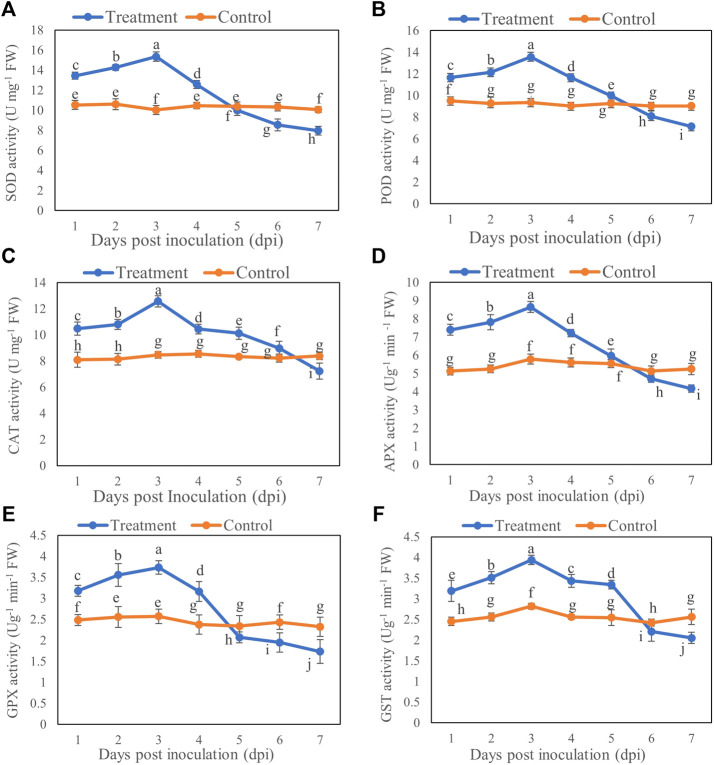
**(A)** SOD, **(B)** POD, **(C)** CAT, **(D)** APX, **(E)** GPX and **(F)** GST activity of *B. brassicae* at different time points after inoculation with *T. longibrachiatum* T6. Different small letters represent the significant difference at the *p <* 0.05 level by Duncan’s new multiple range test. The line bars represent means ± standard error.

The enzyme activity of SOD, POD, CAT, APX, GPX, and GST of *B. brassicae* was increased initially after inoculation with T6 spore suspension compared with the control. The activities of SOD, POD, CAT, APX, GPX, and GST were significantly increased at Day 3 after inoculation compared to those of controls. A significant decrease in all activities was recorded at Day 7 after inoculation compared to control. The activities of SOD, POD, CAT, APX, GPX, and GST showed maximum increase at Day 3 which was 52.98% (Figure 3A), 44.77% (Figure 3B), 48.26% (Figure 3C), 49.39% (Figure 3D), 45.13 (Figure 3E), and 39.67% (Figure 3F) respectively post inoculation compared to control. However, the maximum decrease in the activities of SOD, POD, CAT, APX, GPX, and GST was recorded at Day 7 which was 20.79% (Figure 3A), 21.05% (Figure 3B), 13.75% (Figure 3C), 20.45% (Figure 3D), 25.38% (Figure 3E), and 19.76% (Figure 3F) respectively post inoculation compared to control.

### Effect of *T. longibrachiatum* T6 on H_2_O_2_ and MDA Contents of *B. brassicae*

**FIGURE 4:**
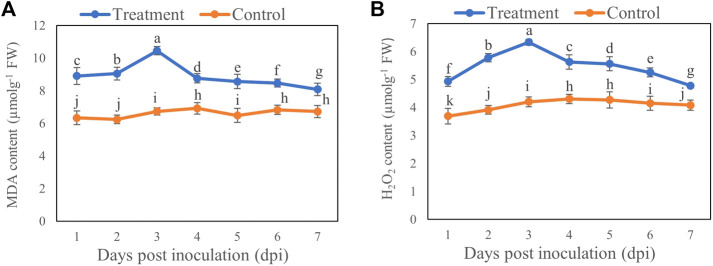
The **(A)** MDA and **(B)** H_2_O_2_ contents of *B. brassicae* at different time points after inoculation with *T. longibrachiatum* T6. Different small letters represent the significant difference at the *p* < 0.05 level by Duncan’s new multiple range test. The line bars represent means ± standard error.

Compared with the control, MDA (Figure 4A) and H_2_O_2_ (Figure 4B) contents were increased by 55.11% and 50.72%, respectively, on Day 3 after inoculation with T6 spore suspension compared with control. However, the MDA and H_2_O_2_ contents showed minimal increase on Day 7 which was 20.14% and 17.01%, respectively, compared to control.

### Effect of *T. longibrachiatum* T6 on Antioxidant Enzyme Genes Expression of *B. brassicae*

**FIGURE 5:**
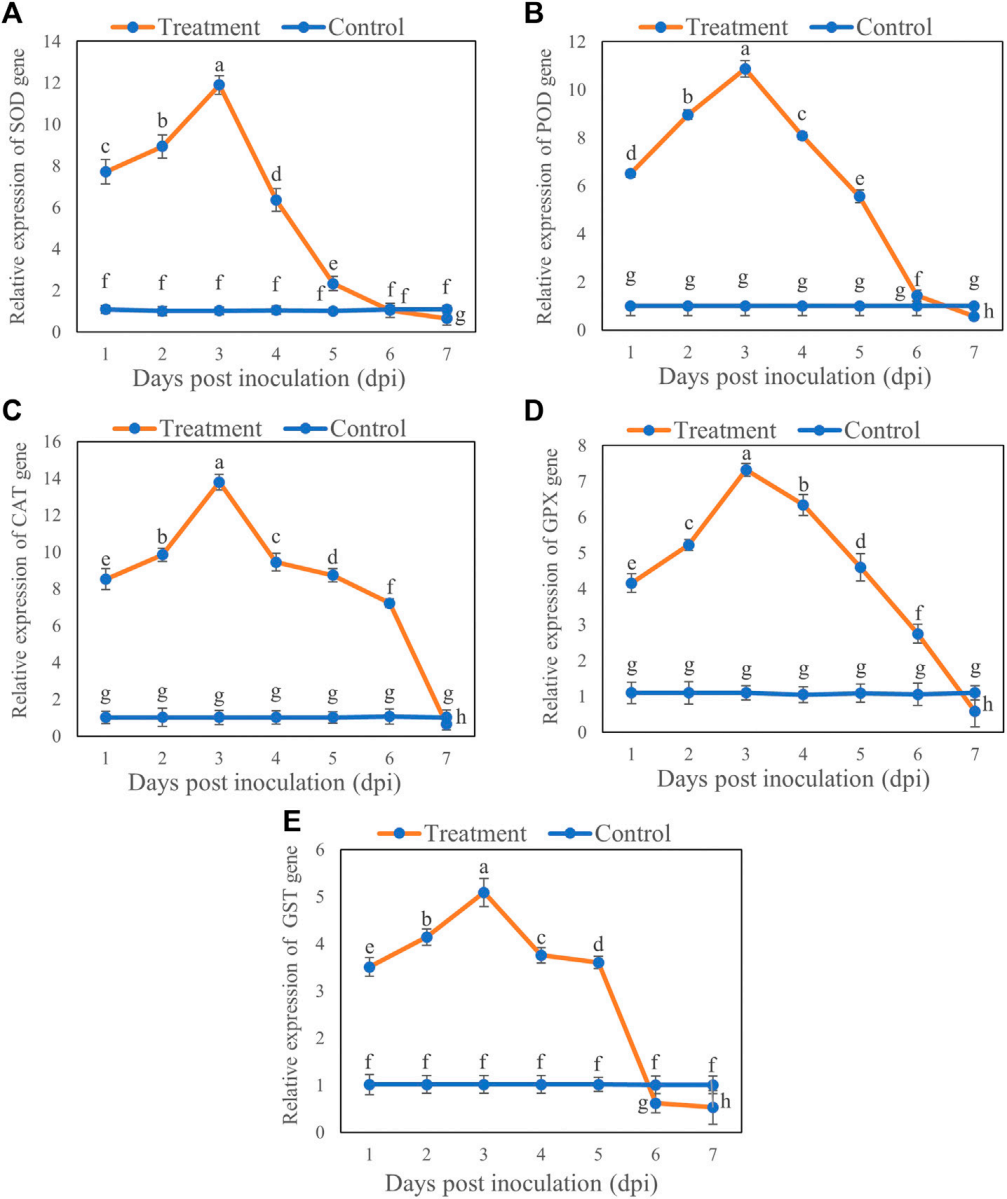
Relative expression of **(A)** SOD, **(B)** POD, **(C)** CAT, **(D)** GPX and **(E)** GST genes in *B. brassicae* at different time points after inoculation with *T. longibrachiatum* T6. Different small letters represent the significant difference at the *p* < 0.05 level by Duncan’s new multiple range test. The line bars represent means ± standard error.

The expression of antioxidant enzyme genes varied at different time points from Day 1 to 7 after inoculation. The expression levels of the genes *SOD*, *POD*, *CAT*, *GPX*, and *GST* were upregulated at first 5 days after inoculation with T6, whereas a significant down regulation was observed at Day 7 compared to control. For all transcription levels of the genes, there was maximum increase in expression levels at Day 3 and then decrease at Day 7. Briefly, the transcription levels of the genes *SOD*, *POD*, *CAT*, *GPX*, and *GST* increased by 10.87 (Figure 5A), 9.87 (Figure 5B), 12.77 (Figure 5C), 6.22 (Figure 5D), and 4.07 (Figure 5E), respectively, on Day 3 after inoculation compared with the control. However, the expression of *SOD*, *POD*, *CAT*, *GPX*, and *GST* genes decreased with increasing duration of inoculation and downregulated at Day 7 compared with the control. The *SOD*, *POD*, *CAT*, *GPX*, and *GST* genes expression were decreased by 0.43 (Figure 5A), 0.44 (Figure 5B), 0.35 (Figure 5C), 0.51 (Figure 5D) and 0.47 at Day 7 (Figure 5E) respectively, compared to control.

### Comparative Expression of Antioxidant Enzyme Genes of *B. brassicae*

**FIGURE 6:**
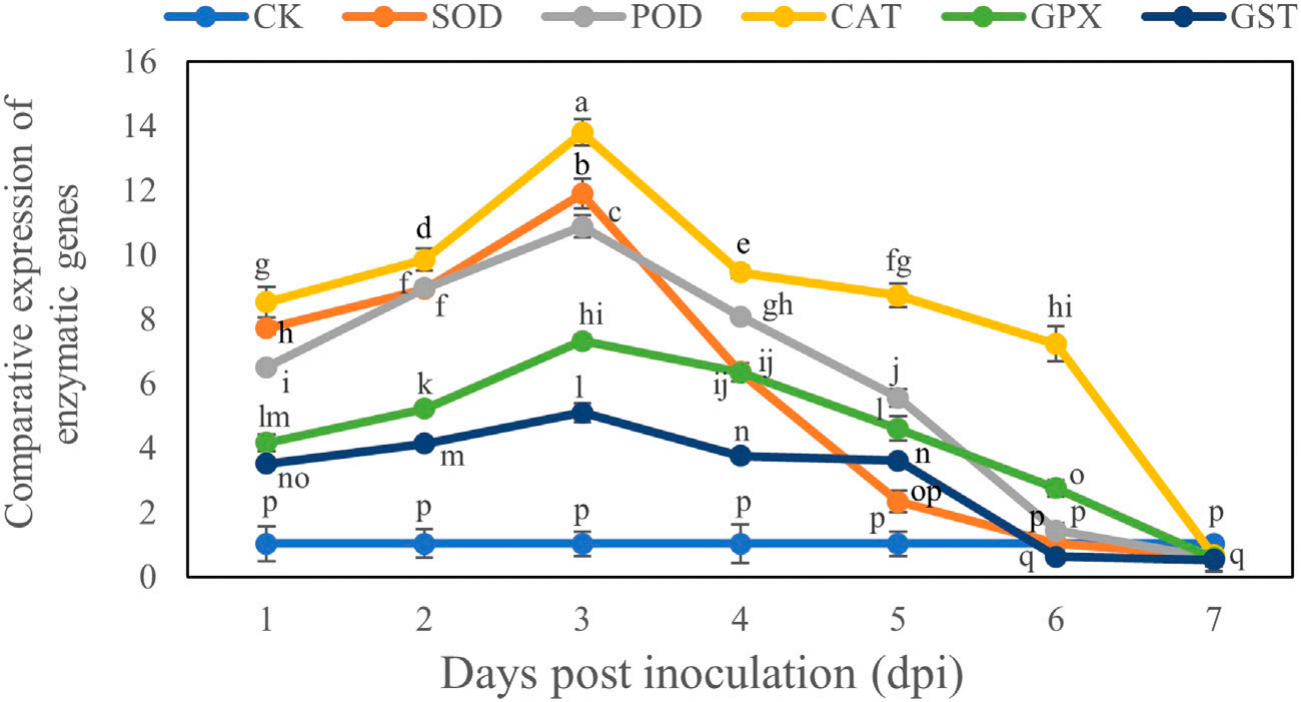
Comparative study of expression of antioxidant enzyme genes in *B. brassicae* inoculated with *T. longibrachiatum* T6 at different time points (1–7 days) post-inoculation. Different letters represent the significant difference at the *p* < 0.05 level by Duncan’s new multiple range test. The line bars represent means ± standard error.

The transcript levels of all genes reached their maximum on Day 3 after inoculation and then declined. The comparative expression levels of all genes showed that the *CAT* gene had the maximum expression level (13.79) on day 3 after inoculation, whereas the *GST* gene had the minimum expression level (5.09) on Day 3 after inoculation compared with the control. However at Day 7 these genes were downregulated and CAT gene decreased by 0.66 and GST gene decreased by 0.53 compared to control at Day 7 (Figure 6).

## Discussion

The present study reported that the strain of *T. longibrachiatum* T6 exhibited significant toxic effect on *B. brassicae*. The highest mortality of *B. brassicae* was observed on Day 7 with a concentration of 1 × 10^8^ spores ml^−1^ after inoculation with T6 spore suspension and the lowest with a concentration of 1 × 10^3^ spores ml^−1^ at day 1. Some previous studies have shown that *Verticillium lecanii* conidia are highly pathogenic to aphids (Fournier and Brodeur, 2000; Kim et al., 2001). In addition, Ansari et al. (2004) found that the mortality of *Hoplia philanthus* varied with the concentration of *M. anisopliae* conidial suspension, exposure time, and temperature. Similarly, several species of coleopteran beetles, such as the coconut palm rhinoceros beetle (*Oryctes rhinoceros*), reached a mortality nearly 100% after 15 days exposure to *Trichoderma* (Nasution et al., 2018), the bean weevil (*Acanthoscelides obtectus*) (Rodríguez-González et al., 2017; Rodríguez-González et al., 2020), and the vineyard borer (*Xylotrechus arvicola*) (Rodríguez-González et al., 2017), also in eggs and larvae (Rodríguez-González et al., 2018).

In a previous study, *Trichoderma* parasitizes insects and obtains nutrients from them to form new conidia on their bodies (Poveda, 2021). *Trichoderma longibrachiatum* and *T. harzianum* parasitized adults of silverleaf whitefly (*Bemisia tabaci*) and tropical bed bug (*Cimex hemipterus*), causing mortality of 40% at 5 days (Anwar et al., 2016) and 90% at 14 days (Zahran et al., 2017), respectively. In the current study, *T. longibrachiatum* T6 (T6) spores attach to the surface of the aphid and infect it by penetrating the cuticle of the aphid, often at the joints or folds where the insect’s protective covering is thinner. Once invaded, it grows by adsorbing nutrients and spreading hyphae throughout the aphid’s body. Infected aphids died due to T6 parasite infestation and a visible color change was observed along with external hyphal growth on the dead body. Our results are in agreement with those of who reported that in fungal infection, the first step before penetration is adhesion of the fungus to the host surface (Dong et al., 2009; Liu et al., 2009). Gabarty et al. (2014) also reported that the entomopathogenic fungi *B. bassiana* and *M. anisopliae* are pathogenic to *Aipsilon larvae* as biocontrol agents.

Low concentrations of ROS are beneficial for signaling between cells and for triggering genes that aid in insect defense (Kamata and Hirata, 1999; Wang, 2001). ROS can lead to oxidative stress when the concentration is high under stressful conditions (Lopez-Martinez et al., 2008). The insects have the ability to eliminate excess ROS from both their internal and extracellular fluids (Krishnan et al., 2007). In this study, the MDA and H_2_O_2_ levels of aphids treated with T6 increased compared to the control group. This might be due to the less efficient activity of detoxifying and oxidoreductive enzymes in the insects, so that the toxic substances secreted by T6 are degraded less effectively. Our results are consistent with those of Ju et al. (2014), who found that the increase in MDA caused by thermal treatment indicates the possibility of damage by ROS accumulation and oxidative stress in *Corythucha ciliata*, since MDA is the endpoint of cell damage in most organisms (Beaulieu and Costantini, 2014). Insects produce enzymes such as SOD, CAT, POD, GPX, and APX, which serve to defend against pathogens and other abiotic stressors (Felton and Summers, 1995). Homayoonzadeh et al. (2022) found that topical application of *B. bassiana* caused 100% mortality in adult *Aphis gossypii* after 7 days because the activities of detoxification enzymes (glutathione S-transferase, carboxylesterase, and acetylcholinesterase) were reduced compared with controls. Digestive enzyme activities were reduced in aphids from inoculated plants, which had higher activity of SOD, APX, and phenoloxidase but lower catalase activity. Energy reserves such as lipids, proteins, and glycogen were lower in aphids of inoculated plants, and they exhibited lower fecundity, longevity, and reproductive duration, as well as a 50% reduction in the LC_50_ of pirimicarb. Thus, inoculation of plants with *B. bassiana* not only caused direct pathogenicity but also negatively affected the physiology and reproductive performance of *A. gossypii*.

Qasim et al. (2021) reported the enzymatic activity and defense-related gene expression of nymphal and adult populations of Asian citrus psyllids (ACP) on Huanglongbing-damaged citrus plants under the attack of *Cordyceps fumosorosea*. A total of five enzymes, namely SOD, POD, CAT, GST, carboxylesterase (CarE), and four genes, namely *SOD*, *16S* gene, *CYP4C68*, *CYP4BD1*, were selected for corresponding observations of ACP populations. The enzymatic activity of four enzymes (SOD, POD, GST, CarE) was significantly decreased after 5 days post-treatment (dpt) and 3 days fungal exposure in fungi-treated adult and nymphal ACP populations, while the activity of CAT was significantly increased after the schedule of treatment. In addition, there were drastic differences in the expression of *CYP4* gene in the fungi-treated ACP populations. At 24 h post-treatment (hpt), the expression of both *CYP4* gene was higher in the fungi-treated populations than in the control populations (adults and nymphs). However, the expression of *CYP4* gene declined in the treated populations after 3 dpt. Similarly, fungal infestation worsened the resistance of the adult and nymphal ACP populations, as the expression of *SOD* was downregulated in the fungi-treated adult and nymphal populations after 5 dpt and 3 dpt of exposure, respectively. Similar to this study, T6 treatment significantly affected antioxidant enzyme activity and gene expression of *B. brassicae*. Inhibition of insect antioxidant activity on the later days caused mortality. In addition, detoxification could be the other crucial adaptive strategy in response to T6 treatment. T6 exposure leads to the expression of several genes involved in detoxification, such as *SOD*, *POD*, *CAT*, *GPX*, and *GST*, which were significantly upregulated across Days (1–3) and stimulated stress response signals in T6-treated aphids. The transcript levels of these genes increased from Days 1 to 3 and decreased sharply from Days 4 to 7 in response to T6 treatment. Therefore, the high mortality rate caused by T6 treatment at Days 4–7 may be due to the inhibition of both enzymatic activity and expressions in *B. brassicae* caused by T6 treatment. Further study may be required to determine the molecular mechanisms of control of *B. brassicae* by T6 both *in vitro* and in field.

## Conclusion

*Trichoderma longibrachiatum* T6 exhibits significant effect in controlling *B. brassicae*, and has the potential to infect *B. brassicae* and affect its antioxidant enzyme activity and gene expression. This study will provide basic knowledge about the effect of fungal stress on aphids and its potential impact on the enzyme activity of the insect, which will be helpful in further studies of T6 in the field experiments. Further field and *in vitro* studies can be carried out to find out the effect and safety of T6 as a safe insecticide against other non-target insects and organisms.

## Data Availability

The raw data supporting the conclusion of this article will be made available by the authors, without undue reservation.

## References

[B1] Ansari M. A. Vestergaard S. Tirry L. Moens M. (2004). Selection of a Highly Virulent Fungal Isolate, *Metarhizium anisopliae* CLO 53, for Controlling *Hoplia philanthus* . J. Invert. Pathol. 85, 89–96. 10.1016/j.jip.2004.01.003 15050838

[B2] Anwar W. Subhani M. N. Haider M. S. Shahid A. A. Mushatq H. Rehman M. Z. (2016). First Record of *Trichoderma longibrachiatum* as Entomopathogenic Fungi against *Bemisia tabaci* in Pakistan. Pak. J. Phytopathol. 28, 287–294.

[B4] Baweja P. Kumar S. Kumar G. (2020). “Fertilizers and Pesticides: Their Impact on Soil Health and Environment,” in Soil Health (New Delhi, India: Springer), 265–285. 10.1007/978-3-030-44364-1_15

[B5] Beaulieu M. Costantini D. (2014). Biomarkers of Oxidative Status: Missing Tools in Conservation Physiology. Conserv. Physiol. 2, cou014. 10.1093/conphys/cou014 27293635 PMC4806730

[B6] Blackman R. L. Eastop V. F. (2008). Aphids on the World's Herbaceous Plants and Shrubs, 2 Volume Set. Chichester, United Kingdom: John Wiley & Sons.

[B7] Boamah S. Zhang S. Xu B. Li T. Calderón-Urrea A. (2021a). *Trichoderma longibrachiatum* (TG1) Enhances Wheat Seedlings Tolerance to Salt Stress and Resistance to *Fusarium pseudograminearum* . Front. Plant Sci. 12, 741231. 10.3389/fpls.2021.741231 34868125 PMC8635049

[B8] Boamah S. Zhang S. Xu B. Tong L. Inayat R. Calderón-Urrea A. (2021b). The Role of *Trichoderma* Species in Plants Response to Salt Stress. Asian J. Res. Crop Sci. 28–43. 10.9734/ajrcs/2021/v6i230114

[B9] Carter C. Sorenson K. (2013). Insect and Related Pests of Vegetables. *Cabbage and Turnip Aphid* . Raleigh, NC, USA: Center for Integrated Pest Management; North Carolina State University.

[B10] Chamzurni T. Oktarina H. Hanum K. (2013). Keefektifan *Trichoderma harzianum* and *Trichoderma virens* Untuk Mengendalikan *Rhizoctonia solani* Kuhn Pada Bibit Cabai (*Capsicum annum* L.). J. Agrista 17, 12–17.

[B11] Charnley A. (1984). “Physiological Aspects of Destructive Pathogenesis in Insects by Fungi: A Speculative Review,” in Symposium Series-British Mycological Society.

[B12] Chivasa S. Ekpo E. Hicks R. (2002). New Hosts of Turnip Mosaic Virus in Zimbabwe. Plant Pathol. 51 (3), 386. 10.1046/j.1365-3059.2002.00699.x

[B13] Dong C. Zhang J. Huang H. Chen W. Hu Y. (2009). Pathogenicity of a New China Variety of *Metarhizium anisopliae* (*M. anisopliae* var. Dcjhyium) to Subterranean Termite *Odontotermes formosanus* . Microbiol. Res. 164, 27–35. 10.1016/j.micres.2006.11.009 17482440

[B15] Durak R. Dampc J. Kula-Maximenko M. Mołoń M. Durak T. (2021). Changes in Antioxidative, Oxidoreductive and Detoxification Enzymes during Development of Aphids and Temperature Increase. Antioxidants 10, 1181. 10.3390/antiox10081181 34439429 PMC8388978

[B16] Felton G. W. Summers C. B. (1995). Antioxidant Systems in Insects. Arch. Insect Biochem. Physiol. 29, 187–197. 10.1002/arch.940290208 7606043

[B17] Fournier V. Brodeur J. (2000). Dose-Response Susceptibility of Pest Aphids (Homoptera: Aphididae) and Their Control on Hydroponically Grown Lettuce with the Entomopathogenic Fungus *Verticillium lecanii*, Azadirachtin, and Insecticidal Soap. Environ. Entomol. 29, 568–578. 10.1603/0046-225x-29.3.568

[B18] Gabarty A. Salem H. M. Fouda M. A. Abas A. A. Ibrahim A. A. (2014). Pathogencity Induced by the Entomopathogenic Fungi *Beauveria bassiana* and *Metarhizium anisopliae* in *Agrotis ipsilon* (Hufn.). J. Radiat. Res. Appl. Sci. 7, 95–100. 10.1016/j.jrras.2013.12.004

[B19] Ghosh S. K. Pal S. (2016). Entomopathogenic Potential of *Trichoderma longibrachiatum* and its Comparative Evaluation with Malathion against the Insect Pest *Leucinodes orbonalis* . Environ. Monit. Assess. 188, 37–7. 10.1007/s10661-015-5053-x 26676413

[B20] Griffin R. Williamson J. (2012). Cabbage, Broccoli and Other Cole Crop Insect Pests. South Carolina: Clemson University. HGIC. *Leucinodes orbonalis* .

[B21] Harman C. Tollefsen K.-E. Bøyum O. Thomas K. Grung M. (2008). Uptake Rates of Alkylphenols, PAHs and Carbazoles in Semipermeable Membrane Devices (SPMDs) and Polar Organic Chemical Integrative Samplers (POCIS). Chemosphere 72, 1510–1516. 10.1016/j.chemosphere.2008.04.091 18614195

[B22] Harman G. E. (2006). Overview of Mechanisms and Uses of *Trichoderma* Spp. Phytopathology 96, 190–194. 10.1094/phyto-96-0190 18943924

[B24] Homayoonzadeh M. Esmaeily M. Talebi K. Allahyari H. Reitz S. Michaud J. P. (2022). Inoculation of Cucumber Plants with *Beauveria bassiana* Enhances Resistance to *Aphis gossypii* (Hemiptera: Aphididae) and Increases Aphid Susceptibility to Pirimicarb. Eur. J. Entomol. 119, 1–11. 10.14411/eje.2022.001

[B25] Jabbar A. S. Sasdoon S. M. (2019). Ecological Studies of Certain Aphid Species and Their Associated Predators on Wheat Plants at Qadisiyah Distract, Iraq. Ind. J. Publ. Health Rese. Dev. 10, 370. 10.5958/0976-5506.2019.00317.6

[B26] Jia M. Cao G. Li Y. Tu X. Wang G. Nong X. (2016). Biochemical Basis of Synergism between Pathogenic Fungus *Metarhizium anisopliae* and Insecticide Chlorantraniliprole in *Locusta migratoria* (Meyen). Sci. Rep. 6, 28424–28515. 10.1038/srep28424 27328936 PMC4916465

[B27] Ju R.-T. Wei H.-P. Wang F. Zhou X.-H. Li B. (2014). Anaerobic Respiration and Antioxidant Responses of *Corythucha Ciliata* (Say) Adults to Heat-Induced Oxidative Stress under Laboratory and Field Conditions. Cell Stress Chaperones 19, 255–262. 10.1007/s12192-013-0451-x 23943359 PMC3933624

[B28] Kamata H. Hirata H. (1999). Redox Regulation of Cellular Signalling. Cell. Signal. 11, 1–14. 10.1016/s0898-6568(98)00037-0 10206339

[B29] Kessing J. Mau R. (1991). Cabbage Aphid, Brevicoryne brassicae *(Linnaeus). Crop Knowledge Master* . Honolulu, Hawaii: Department of Entomology. (2 October 2013).

[B30] Khachatourians G. (1986). Production and Use of Biological Pest Control Agents. Trends Biotec. 4, 120–124. 10.1016/0167-7799(86)90144-7

[B31] Khurshid A. Inayat R. Tamkeen A. Ul Haq I. Li C. Boamah S. (2021). Antioxidant Enzymes and Heat-Shock Protein Genes of Green Peach Aphid (*Myzus persicae*) under Short-Time Heat Stress. Front. Physiol. 12, 805509. 10.3389/fphys.2021.805509 34975546 PMC8718642

[B32] Kim J. J. Lee M. H. Yoon C.-S. Kim H. Yoo J.-K. Kim K.-C. (2001). Control of Cotton Aphid and Greenhouse Whitefly with a Fungal Pathogen. Bio. Cont. Greenhouse Pests, 8–15.

[B33] Krishnan N. Kodrík D. Turanli F. Sehnal F. (2007). Stage-specific Distribution of Oxidative Radicals and Antioxidant Enzymes in the Midgut of *Leptinotarsa decemlineata* . J. Insect Physiol. 53, 67–74. 10.1016/j.jinsphys.2006.10.001 17126855

[B34] Krishnan N. Sehnal F. (2006). Compartmentalization of Oxidative Stress and Antioxidant Defense in the Larval Gut of *Spodoptera littoralis* . Arch. Insect Biochem. Physiol. 63, 1–10. 10.1002/arch.20135 16921519

[B35] Liu W. Xie Y. Xue J. Gao Y. Zhang Y. Zhang X. (2009). Histopathological Changes of *Ceroplastes japonicus* Infected by *Lecanicillium lecanii* . J. Inver. Pathol. 101, 96–105. 10.1016/j.jip.2009.03.002 19306882

[B36] Livak K. J. Schmittgen T. D. (2001). Analysis of Relative Gene Expression Data Using Real-Time Quantitative PCR and the 2−ΔΔCT Method. Methods 25, 402–408. 10.1006/meth.2001.1262 11846609

[B37] Lopez-Martinez G. Elnitsky M. A. Benoit J. B. Lee R. E. Jr Denlinger D. L. (2008). High Resistance to Oxidative Damage in the Antarctic Midge *Belgica antarctica*, and Developmentally Linked Expression of Genes Encoding Superoxide Dismutase, Catalase and Heat Shock Proteins. Insect Biochem. Mol. Biol. 38, 796–804. 10.1016/j.ibmb.2008.05.006 18625403

[B40] Lwetiojera D. W. Sumaye R. D. Madumla E. P. Kavishe D. R. Mnyone L. L. Russell T. L. (2010). An Extra-domiciliary Method for Delivering Entomopathogenic Fungi, *Metarhizium Anisopliae* IP 46 against Malaria Vectors, *Anopheles arabiensis* . Parasites Vectors 3. 10.1186/1756-3305-3-18 PMC2848008 20233423

[B41] Mnyone L. L. Kirby M. J. Lwetoijera D. W. Mpingwa M. W. Knols B. G. Takken W. (2009). Infection of the Malaria Mosquito, *Anopheles gambiae*, with Two Species of Entomopathogenic Fungi: Effects of Concentration, Co-formulation, Exposure Time and Persistence. Malar. J. 8, 309–312. 10.1186/1475-2875-8-309 20030834 PMC2808315

[B43] Munthali D. C. (2009). Evaluation of Cabbage Varieties for Resistance to the Cabbage Aphid. Afr. Entomol. 17, 1–7. 10.4001/003.017.0101

[B44] Nasution L. Corah R. Nuraida N. Siregar A. Z. (2018). Effectiveness *Trichoderma* and *Beauveria bassiana* on Larvae of *Oryctes rhinoceros* on Palm Oil Plant (Elaeis Guineensis Jacq.) *In Vitro* . Int. J. Environ. Agric. Biotec. 3, 239050. 10.22161/ijeab/3.1.20

[B45] Opfer P. Mcgranth D. (2013). Oregon Vegetables, Cabbage Aphid and Green Peach Aphid. Corvallis, OR. Dispon: Department of Horticulture. Oregon State University.

[B46] Poveda J. (2021). *Trichoderma* as Biocontrol Agent against Pests: New Uses for a Mycoparasite. Bio. Control 159, 104634. 10.1016/j.biocontrol.2021.104634

[B47] Qasim M. Xiao H. He K. Omar M. A. A. Hussain D. Noman A. (2021). Host-pathogen Interaction between Asian Citrus Psyllid and Entomopathogenic Fungus (*Cordyceps Fumosorosea*) Is Regulated by Modulations in Gene Expression, Enzymatic Activity and HLB-Bacterial Population of the Host. Comp. Biochem. Physiol. Part C Toxicol. Pharmacol. 248, 109112. 10.1016/j.cbpc.2021.109112 34153507

[B48] Rodríguez-González Á. Carro-Huerga G. Mayo-Prieto S. Lorenzana A. Gutiérrez S. Peláez H. J. (2018). Investigations of *Trichoderma* Spp. And *Beauveria bassiana* as Biological Control Agent for *Xylotrechus arvicola*, a Major Insect Pest in Spanish Vineyards. J. Econ. Entomol. 111, 2585–2591. 10.1093/jee/toy256 30165386

[B49] Rodríguez-González Á. Campelo M. P. Lorenzana A. Mayo-Prieto S. González-López Ó. Álvarez-García S. (2020). Spores of *Trichoderma* Strains Sprayed over A*canthoscelides obtectus* and *Phaseolus vulgaris* L. Beans: Effects in the Biology of the Bean Weevil. J. Stored Prod. Res. 88, 101666. 10.1016/j.jspr.2020.101666

[B50] Rodríguez-González Á. Mayo S. González-López Ó. Reinoso B. Gutierrez S. Casquero P. A. (2017). Inhibitory Activity of *Beauveria bassiana* and *Trichoderma Spp*. On the Insect Pests *Xylotrechus arvicola* (Coleoptera: Cerambycidae) and *Acanthoscelides obtectus* (Coleoptera: Chrisomelidae: Bruchinae). Environ. Monit. Assess. 189, 1–8. 10.1007/s10661-016-5719-z 27933578

[B51] Sparagano O. A. E. Giangaspero A. (2011). “Parasitism in Egg Production Systems: the Role of the Red Mite (*Dermanyssus gallinae*),” in Improving the Safety and Quality of Eggs and Egg Products (Ottawa, ON, Canada: Elsevier), 394–414. 10.1533/9780857093912.3.394

[B52] Srinivasan R. (2008). Integrated Pest Management for Eggplant Fruit and Shoot Borer (*Leucinodes Orbonalis*) in South and Southeast Asia: Past, Present and Future. J. Biopest. 1, 105–112.

[B53] Valko M. Rhodes C. J. Moncol J. Izakovic M. Mazur M. (2006). Free Radicals, Metals and Antioxidants in Oxidative Stress-Induced Cancer. Chemico-Biological Interact. 160, 1–40. 10.1016/j.cbi.2005.12.009 16430879

[B54] Wang J. (2001). Glucose Biosensors: 40 Years of Advances and Challenges. Electroanalysis 13, 983–988. 10.1002/1521-4109(200108)13:12<983::aid-elan983>3.0.co;2-#

[B56] Zahran Z. Mohamed Nor N. M. I. Dieng H. Satho T. Ab Majid A. H. (2017). Laboratory Efficacy of Mycoparasitic Fungi (*Aspergillus tubingensis* and *Trichoderma harzianum*) against Tropical Bed Bugs (*Cimex hemipterus*) (Hemiptera: Cimicidae). Asian Pac. J. Trop. Biomed. 7, 288–293. 10.1016/j.apjtb.2016.12.021

[B57] Zhang S. Gan Y. Xu B. Xue Y. (2014). The Parasitic and Lethal Effects of *Trichoderma longibrachiatum* against *Heterodera avenae* . Biol. Control 72, 1–8. 10.1016/j.biocontrol.2014.01.009

